# Social Innovation in Home-Based Eldercare: Strengths and Shortcomings of Integrating Migrant Care Workers into Long-Term Care in Tuscany

**DOI:** 10.3390/ijerph191710602

**Published:** 2022-08-25

**Authors:** Marlene Seiffarth, Giulia Aureli

**Affiliations:** SOCIUM, University of Bremen, 28359 Bremen, Germany

**Keywords:** social innovation, long-term care, home-based care, migrant care workers, Italy, Tuscany

## Abstract

Italy is one of the main receiving countries of migrant care workers in Europe. Its migrant-in-the-family model has developed since the 1990s, and, today, home-based eldercare is unimaginable without the work of the almost one million care workers employed in private households, of whom over 75% are migrants. Despite forming one of the most important pillars of eldercare provision in the country, the employment of migrant care workers is not addressed in national policy. However, regional policymaking is far from inactive in the face of growing gaps in care as regions and municipalities play a crucial role in regulating, organising, and providing eldercare. With a focus on comprehensive solutions, cross-sector collaborations, and interactive learning processes, social innovation becomes an important element in reforming eldercare in the context of institutional inertia, fragmentation, and permanent austerity. In what ways are regions using social innovation to respond to challenges in eldercare provision and integrate migrant care workers? This study is based on interviews with experts from the region of Tuscany, which is running the project Pronto Badante (emergency care worker). The results suggest several advantages of local interventions breaking with the institutional silo mentality, as well as ongoing challenges regarding the impact and sustainability of these interventions.

## 1. Introduction

The COVID-19 outbreak in Italy at the beginning of the pandemic exposed the ongoing care crisis in the country. The country’s residential care facilities became infamous for their lack of adequate response in the emergency and the high number of older persons who lost their lives to the virus [1]. However, public provision of eldercare in residential care homes and through homecare services represents only a fraction of the overall care provided for older people in the country. In Italy’s familialistic welfare regime, it is family members who shoulder the bulk of care work [2,3,4,5]. Alongside the estimated 3.3 million unpaid family caregivers [6], the largely unregulated sector of family assistants (badanti), directly hired by Italian households, represents the other important pillar of long-term care (LTC) provision. There are 437,663 family assistants providing LTC in private households, of whom 92% are women and 73% are migrant care workers (MCWs) [7]. This figure more than doubles to over one million family assistants when including the estimated 57% of those in informal employment, that is, without social security registration [8].

Over the last three decades, research on migrant care work has become prominent in several social-policy-related fields, such as LTC, migration, and domestic work. This research concerns the employment of migrants primarily in private households in industrialised countries who care for older adults in need of support in their daily lives. The high incidence of migrant care work in Italy has resulted in numerous studies in various disciplines focussing on issues such as irregular migration and care chains [9,10,11,12,13]. Moreover, the country has also been frequently included in comparative studies on welfare and care regimes [3,5,14,15,16,17,18]. The main findings of this body of literature are Italy’s ‘transition from a “family” to a “migrant in the family” model of care’ [19] (p. 272) and the absence of reform in the country’s LTC sector (e.g., Ref. [20]). These issues are interconnected and produce challenging outcomes.

The difficulties in Italy’s public and private care arrangements are linked to its demographic challenges—faced by all European countries—regarding population ageing, such as the shrinking of the overall population, increasing life-expectancy, and rising dependency ratios [21]. To address these challenges, the call for more social innovation in LTC policies and practices has become evident in LTC literature [22,23,24]. According to Heinze and Naegele [25], ‘population ageing can be regarded as both drivers for social change as well as [the] point of departure for social innovations which aim at tackling with its challenges’ [25] (p. 155). This point of departure for social innovation is particularly compelling in Italy, which not only has one of the oldest populations of OECD countries, but, by 2050, it is estimated that one-third of its population will be aged 65 and above, and almost 15% 80 and older [26]. Moreover, Italy’s socio-political context of stagnant LTC policies, fragmented and regionally diverging public eldercare provision, as well as a sizable and largely unregulated market of MCWs, provide ample challenges relevant to socially innovative practices.

Using the region of Tuscany and its project Pronto Badante (emergency care worker) as a case study, this article examines the following research question: *in which ways is the region of Tuscany using social innovation to respond to challenges in LTC provision and to integrate MCWs?*

This paper starts by outlining our theoretical framework, providing context regarding LTC in Italy (Section 2), and presenting our research design (Section 3). The results from the expert interviews we conducted will be presented and analysed thereafter: we provide an overview of Pronto Badante (Section 4) and then assess the project’s social innovative character (Section 5), as well as its strengths and shortcomings of integrating MCWs (Section 6 and Section 7). This analysis is inspired by the findings of Schulmann, Reichert, and Leichsenring [22], who identified key drivers and barriers to social innovation in LTC by examining 60 initiatives across 11 countries (including Italy). Our findings are discussed throughout these sections before we conclude with a summary and policy recommendations.

## 2. Social Innovation in Long-Term Care in Italy and its Regions

### 2.1. A Systematic Approach to Social Innovation

Most scholars agree that social innovation has become a buzzword based on vague and varying definitions, which has led some to call it a precept, or a ‘theoretical and practical work-in-progress’ [27]. The term social innovation has become increasingly popular in the 2010s, but its use is not without history. In fact, innovation took on positive connotations only in the 19th century [28]. In a broad sense, its recent popularity is linked to this linguistical turn, but, in a narrow sense and with a view towards social policy, the introduction of the European Commission’s impetus on social innovation in 2010 and its subsequent funding of projects have had an undeniable impact [29]. The European Commission uses the following definition:

*Social innovation is about new ideas that work to address pressing unmet needs. We simply describe it as innovations that are both social in their ends and in their means. Social innovations are new ideas (products, services, and models) that simultaneously meet social needs (more effectively than alternatives) and create new social relationships or collaborations.*

(EC, 2010)

We follow scholars in LTC research who are using this definition in a pragmatic fashion to evaluate policy interventions and projects. Madama et al. [29] point out:

*what seems to emerge from empirical research is the opportunity to increase the denotative power of the concept of ‘social innovation’ by adopting operational definitions at a lower ladder of abstraction, which, on the one hand, allows the capture of different degrees of innovation and, on the other hand, takes into account the specificities of the policy and the welfare context in which solutions that can be qualified as ‘innovative’ are located.*

[29] (p. 129)

Following this pragmatic stance on operationalising social innovation for empirical work, our research leans towards the practical rather than the critical stream of social innovation scholarship. Moulaert and MacCallum [27] distinguish these two streams but note that there are overlaps and continuums. Our analysis of cross-sectoral collaborative arrangements and public sector innovation, as well as of effective service delivery [27] (p. 45), focusses on identifying promising solutions in these regards. However, in centring marginalised members of society and the labour market, such as MCWs, we keep the underlying structures and inequalities of globalised neoliberalism in mind and see social innovation as an antidote to purely market-driven solutions.

Although social innovations evolve and advances in technology have been immense in the past decade, the framework for evaluating social innovation proposed by Heinze and Naegele [25] is still valuable for our purposes. In conjunction with the complex challenges of migrant care work, their focus on comprehensive solutions, cross-sector collaborations, and interactive learning processes are crucial, especially for the field of LTC. They outline preconditions that must be met to speak of social innovation, and these form the basis for our systematic evaluation (see Section 5).

### 2.2. Italian Long-Term Care

In Italy, LTC has remained a residual aspect of social welfare and is characterised by outright policy inertia as there have been no national reforms that substantially addressed the current LTC arrangement [16,20,30,31]. Thus, Italian LTC continues to be funded and regulated in different policy fields via national, regional, and local institutions, leading to an institutional fragmentation due to the multitude of heterogeneous actors involved. With reforms of Italy’s tax-funded National Health Service (NHS) in the 1990s and a new framework law passed on social services in 2000, regions were delegated more responsibility in the management and delivery of health and social services [32]. Regions have since been responsible for regulating access to services and determining how the various actors of the local system relate to each other [33]. In response, the national government, through constitutional reform in 2001, reserved the right to determine national standards to safeguard basic benefit levels, thereby strengthening multilevel governance [32]. On the one hand, this heightened even more the regional disparities in the country regarding access to and quality of public services, including LTC services [34,35]. In fact, most issues regarding public LTC provision are dealt with regionally and municipally [23,36]. On the other hand, LTC follows a pattern of ‘vicious layering’ since this ‘multilevel governance arrangement [is one] whose design encourages the dysfunctions (gaps and overlaps, inefficiencies, blame and cost shifting, and territorial inequalities) while it limits the benefits of decentralization’ [37] (p. 652). As shown by Arlotti and Aguilar-Hendrickson [37], the key elements of vicious layering can be found in LTC governance in Italy. The result is serious dysfunction in the provision of LTC. In turn, the increasing demand for eldercare cannot be met by public services.

In addition to policy inertia, institutional fragmentation, and vicious layering, permanent austerity has also limited investment in public care infrastructures [38]. Not surprisingly, albeit unintentionally, cash-for-care schemes (both at the national and regional level) have become the only growing elements of Italian LTC [39]. Consequently, while Italy’s LTC expenditure is exactly the EU average of 1.7% [40], around half of this expenditure is spent on the cash-for-care scheme Indennità di Accompagnamento, which is a non-means-tested monetary benefit provided at the national level to fully dependent citizens. This leaves little room for maintenance, let alone investment in public LTC infrastructure [41]. The presence of other similar benefits at the regional level, mostly means-tested, further underline the importance of monetary benefits in Italian LTC [6]. The dysfunction of the public system and its focus on monetary benefits have led to LTC dominated by family caregivers and MCWs [14]. As an illustration, the estimated 2.1 million domestic and care workers in Italy who support families with their care needs [8] represent more than three times the number of all NHS workers combined (649,517 in 2019) [42].

### 2.3. Social Innovation in Practice: Regional Social Innovations in Italian Long-Term Care

As a testing ground for new ideas, the local governance level is often the one experimenting with and implementing social innovations [43]. Since LTC in Italy is mainly carried out by regional and local actors, it is not surprising that social innovation in LTC is brought forward by regions rather than the national government. Due to little political will and lack of capacity to reform or invest in LTC at the national level, regions often resort to funding opportunities from the EU, which has been promoting a policy framework that focusses on ageing, LTC, and social innovation, including funding opportunities for local initiatives, since the late 2000s [33,44].

Informed by a few academic publications and by screening the grey literature, we see some general trends of social innovation in practice. First, although EU funding is sought after by many Italian regions to compensate for the lack of public spending in several policy fields, interventions in LTC seem to be concentrated in Italy’s Northern and Central regions, especially in Lombardy, Tuscany, and Piedmont, but also in Emilia-Romagna and Marche [29,33,45]. Second, many innovative interventions mainly address shortcomings of the public LTC components, such as homecare services and day care centres (residential care homes to a lesser extent), at the expense of LTC provided in the family setting. These interventions often concern the prevention or delay of institutionalisation (as the most expensive element of LTC), in line with the concept of ‘ageing in place’, which is a prominent topic in the promoted EU strategies concerning LTC [33]. Bottom-up initiatives often create multi-stakeholder networks to provide for conditions enabling dependent older people to remain in their own homes as long as possible by involving paid professionals but also volunteers [29]. For example, family nurse programmes, such as in Lombardy, aim to provide direct and technical assistance to people with chronic illnesses and to improve the response of the local healthcare services via the creation of interdisciplinary teams [45]. In Piedmont, projects focused on the promotion of autonomy and homecare use local social services, specialised medical staff, as well as volunteers to respond to older persons in need and foster their community involvement [29]. Third, another set of interventions are focused on knowledge and skills, especially to support family caregivers, sometimes family assistants, to educate carers on how to deal with specific pathologies or to certify informally acquired skills. The use of information and telecommunications technologies (ICT) is prominent among these projects and includes e-learning for training, telemedicine for treatment, GPS location for tracking dementia patients, or apps/websites for communication between the welfare system and citizens [33,46].

In practice, social innovation in Italian regions concerned with addressing both the gaps in governance of LTC, as well as the shortcomings of LTC provision, seems to neglect the integration of MCWs [45] despite their vital role in enabling older people’s ‘ageing in place’. Paid family assistants (of whom the majority are MCWs) compensate for decreasing resources of family caregivers and insufficient public homecare [47], and the deep-rooted informality and precarity of their employment calls for innovative solutions.

## 3. Materials and Methods

### 3.1. Case Selection

In their analysis of social innovation in LTC in Italy, Casanova et al. [45] underline the urgency of social innovation needed in homecare, especially innovation that addresses user needs, new organisational and governance models, and prevention activities. The Tuscan project Pronto Badante, recently identified as one of the few successful projects in the country [48], covers two of these three categories of urgently needed social innovation. First, the project integrates mixed services to support family care and MCWs, as well as establishing case management. Second, it creates new collaborations and a multi-stakeholder network.

This project was initiated by the regional council of Tuscany, a region where the migrant-in-the-family model is firmly established. Arguably, the model is in its most original form when MCWs cohabit with care-receivers. Of all Italy’s regions in 2019, the share of this live-in care among all domestic workers was highest in Tuscany (56%), whereas the national average was 31%. Ninety-four percent of these live-in workers are migrants [49]. Moreover, the region also has an above-average number of people 75 and older (14% compared to the national average of 11%). From these factors, it becomes clear that the case of Tuscany was selected as an extreme case [50]. In other words, it is not representative of the country and, therefore, has the potential to provide insights for policymakers in other regions. Hence, we systematically evaluate the Tuscan intervention Pronto Badante and highlight possibilities for regional policymaking on integrating MCWs into the local LTC systems.

### 3.2. Data Collection and Analysis

This paper is based on secondary and grey literature, as well as 10 semi-structured interviews with 13 experts from Tuscany (see Table 1), which were conducted in Italian using online videoconferencing. In two of these interviews, more than one expert was present. Prior to the interviews, all participants received information on consent and data protection and then signed informed consent forms, in which they also agreed to being quoted in publications. The first round of interviews took place in April 2021 and was targeted at representatives of the regional public administration, and regional and local non-profit organisations (NPOs), mainly social cooperatives, connected to Pronto Badante. These interviews were 50–110 min in length and were transcribed. In the second round, organisations not a part of the project were targeted (indicated in Table 1 by *). Four short expert interviews (30–60 min) took place in September 2021 and were then summarised. The main questions in all interviews referred to Tuscany’s policies and interventions regarding dependent older people and MCWs, respectively, as well as the effects of the COVID-19 pandemic on the family assistance sector. Whereas we immediately inquired about details on Pronto Badante in the first round, we did not mention the project in the second round until it was brought up by the interviewees themselves. All interview material was subject to a content analysis focused on inductively identifying themes and issues regarding an assessment of Pronto Badante and regional interventions on migrant care work.

## 4. Overview of Pronto Badante

*[Let’s say] I’m an old man of 80 years, I’m fine, [but then] I fall, I break my femur, I go to the hospital, they take me in, they operate, they do everything they have to do, and then they tell me you can’t stay here because there’s no room for the other [patients]. At that point the family comes into play, but how can the family face this emergency when it has no expertise?*

(NPO-1)

This vivid scenario provided as an example by an interviewee describes a common problem in all Italian regions: older persons and their families are ignored in situations of emerging LTC needs. The central region of Tuscany is not immune to this challenge, as well as the connected issues of overcoming vicious layering of governance and informal employment of MCWs. This section describes Tuscany’s solution to these common problems.

Although interventions in homecare in other regions share some of Pronto Badante’s features [45], it is the only project of its kind in the country according to the interviewees (as of March 2022). The idea for the project grew out of the needs of citizens, as identified by Councillor Stefania Saccardi, who had just moved from the municipal to the regional government. The lawyer was, inter alia, deputy mayor and welfare councillor of the city of Florence, the region’s most populated municipality and home to many older people living on their own. During her first term as vice president of the Tuscany Regional Council from 2013 and 2015, she addressed the concerns of citizens and civil society organisations (e.g., pensioners’ unions) by launching Pronto Badante in October 2014 (NPO-1; PA-1a).

Like Pronto Soccorso, the rapid response of emergency rooms in hospitals, Pronto Badante was also designed to ease a crisis but one focused on home-based eldercare and integrating MCWs (NPO-1). It does so with the promise of a home visit by a social worker within 48 h of the crisis being identified. The target population is people over 65 years old who have not previously received LTC. First, people reach a call centre using a region-wide toll-free hotline and present their case. The hotline is open with extended hours to make it accessible to most people (Monday to Friday: 8 a.m.–7.30 p.m.; Saturdays: 8 a.m.–3 p.m.) (PA-1a). Callers either receive information or they are directed to a case manager in their municipality, who schedules a home visit. During the home visit, the case manager provides support to the person in need and their family members: they ‘*go to the home and become aware of the situation, […] take the family and the elderly person by the hand and give them information on what services are available in the area*’ (PA-1a). In most cases, the goal is to find a family assistant, usually an MCW (due to the high number of migrants in this sector), who can be paid with a service voucher. Once the family has found an MCW, they contact the case manager, who then takes care of the paperwork to issue the voucher. The project makes use of the family booklet, a pre-funded instrument of payment vouchers each worth 10 Euro, funded by the INPS, which was intended to pay for occasional babysitting, tutoring, and domestic help. The voucher totals 300 Euro (30 h of care work at 10 Euro per hour provided by a family assistant). Since family caregivers have access to benefits through other channels, a decision was made that the benefit would be in the form of wages for care workers, not a cash benefit for families. The advantage of such a voucher is its conditionality: families can only use the voucher for services of care workers who are formally employed, that is, with a contract and social security registration. This represents a conscious effort on the part of Pronto Badante to address the widespread informal employment of MCWs. Although the case manager may facilitate the search for a family assistant by making suggestions on where to find an MCW, the project itself does not recruit or match worker to family. Rather, the process of finding family assistants is context-specific. However, some organisations involved with Pronto Badante have set up databases of suitable candidates for family assistance.

Some of the social cooperatives that are a part of Pronto Badante have created short training courses for MCWs. In the municipality of Prato, for example, a social cooperative has created a basic 40-h training course, of which 30 h are theoretical and 10 are on-the-job training. This course is intended to establish a baseline qualification for the MCWs whom they license (NPO-3). In other areas, the MCWs who complete training are added to an organisation’s database for matching families with workers. Another cooperative has shifted its training course into an online format, which has been incorporated into the portfolio of Pronto Badante and is now available to MCWs across the region regardless of where they live (NPO-2).

**Figure 1:**
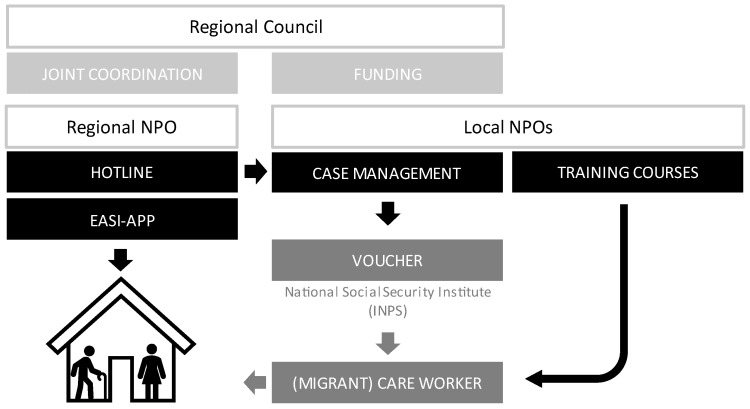
Organisational overview of Pronto Badante. Source: authors’ compilation based on expert interviews.

Organisationally, the regional coordination team of Pronto Badante has staff from the regional council and Esculapio, a regional non-profit organisation (NPO), which runs the toll-free hotline and provides annual training sessions for case managers. The case management is run by local NPOs (see Figure 1 for an organisational chart of Pronto Badante). Each year, the regional council invites tenders from local organisations for monies that would fund their social workers as the project’s case managers. In addition to authorising the INPS vouchers for MCWs, the case managers can also authorise the services of physiotherapists, nurses, psychologists, and volunteers. These providers are based at the local NPOs carrying out Pronto Badante or other local organisations. The following sections provide more detailed information about the different elements of the project.

In quantitative terms, the project had the following results in the period 2016–2021 (information received by administrator of the project on 6 August 2021):

1. More than 81,000 calls to the toll-free hotline;
2. Almost 26,000 visits made to elderly persons’ homes;
3. More than 18,700 vouchers issued for employed MCWs.

## 5. Pronto Badante as Social Innovation Good Practice?

In their descriptions of Pronto Badante, several interviewees described the project as both experimental and innovative: ‘*a small but in the end also a large laboratory of research, of welfare innovation*’ (PA-1a) and ‘*it has been possible year by year to experiment with innovative methods of intervention*’ (NPO-1). The experimental qualities relate mostly to the project’s temporary and provisional nature. It was begun in 2014 as a pilot project in the province of Florence, followed by an experimental phase (2016–2019) and a stabilisation phase (since 2019) in the entire region with annual renewals.

The innovative qualities of Pronto Badante were highlighted by those experts directly involved in either the coordination or the evaluation of the project. Interviewees highlighted its novelty and uniqueness without referring to it as ‘innovative’. This might be connected to the findings of Casanova et al. [45], who observed that the term social innovation is either linked to technological innovations or not used by stakeholders in the field of LTC.

Considering the urgent need for new and innovative approaches in Italian LTC and the claims by those involved in Pronto Badante, evaluating the quality of its social innovativeness is appropriate. Appraisals of social innovations in LTC often draw on Heinze and Naegele’s [25] preconditions that must be met for social innovation to have occurred, which we adhere to as well. The project fulfils eight out of eleven preconditions, and, therefore, we do not consider it as social innovation per se but a project with social innovative qualities (see Table 2 for a summary of our assessment of Pronto Badante).

## 6. Strengths

### 6.1. At-Home Assistance and Integrating Migrant Care Workers

Pronto Badante, with its focus on rapid responses to emerging LTC needs, is oriented towards the pressing challenge of care gaps in LTC, particularly those around the onset of LTC needs. The crucial elements of change that Pronto Badante addresses are the time and the place of the intervention as ‘*the public system in reality did not guarantee quick interventions and at home, and it is on these two elements that Pronto Badante brought the change, that is, it provided those in need with something that met their needs in a short time and, above all, at their homes*’ (PA-1b). Users are positively surprised about being attended to in their own homes, and case managers are able to see for themselves what kind of support is needed during these home visits.

Another social issue the project addresses is the employment of MCWs, which is usually informal, meaning without social security contributions. The project aims to create new configurations of employment by making access to the financial INPS voucher conditional on formally hiring MCWs, unlike other vouchers and benefits on which the type of employment of care workers has no bearing. Due to high levels of informality in the domestic work sector, promoting its formalisation is of utmost importance since the benefits achieved for domestic workers by collective agreements are only available to those in formal employment [51,52]. Moreover, most of the MCWs hired by families receiving assistance from Pronto Badante are not trained care workers, so the training courses offered by the participating local organisations and the digital courses are vital in the professionalisation of the sector. Both formalisation and professionalisation are also a priority for national trade unions and associations of employers of domestic workers [52].

### 6.2. New Forms of Relations and Collaborations

Pronto Badante brings together stakeholders of the public and third sector that provide homecare. Instead of working in tandem, the project has sought to use the strengths of the third sector as the bridge between the public sector and the family assistance sector. With its target on MCWs, the project also attempts to connect the public and the third sector, as well as the third sector and the unregulated sector of migrant care work. However, these efforts were initially met with resistance. Public social services were apprehensive about Pronto Badante as they feared a surge of referrals to their services, which already operated at maximum capacity. However, the number of dire cases handled by Pronto Badante that have been transferred to social services has remained low. Instead, the project has led to more informed and collaborative relationships between the public and the third sector and appears to have overcome the silo mentality common across Italian health and social services institutions. Now, public social services can even bridge staff shortages by referring cases to local organisations carrying out Pronto Badante’s mandate (PA-1b). The third sector was also apprehensive at first about the unregulated market of MCWs. Social cooperatives in particular perceived Pronto Badante to be intent on organising the migrant care work market, which would have directly competed with their provision of home-based eldercare. However, these fears did not materialise (NPO-2).

Another set of important stakeholders in the project are the organisations of social partners, which are instrumental in setting up contracts and managing payrolls for family employers [52]. A local trade union representative called Pronto Badante a ‘*proactive vortex*’ and stressed the synergetic problem-solving qualities of the project as people who called the hotline or were visited at home were referred to local trade unions (TU*). However, a representative of a local family employers’ association did not share this impression of referrals from the project to their association (EA*).

### 6.3. Resilience in Pandemic Times through Technology

During the COVID-19 pandemic, Pronto Badante provided uninterrupted assistance. While home visits were suspended during the strict lockdown, they were replaced by telephone calls. Moreover, families were able to take advantage of the new technologies developed within the project even prior to the pandemic. The application EASI (E-Assistant for Senior Improvement) allows for video calls between the older person and the Pronto Badante case manager to offer support and remote contact to ease feelings of isolation. Pre-pandemic, the app was used via a web browser and had been made available to a group of chosen test users who were given tablets for trial usage. In spring 2020, the app became downloadable on any smartphone and, hence, available to anyone precisely when it was urgently needed (NPO-3). The app’s video call feature is exclusive to participants registered with Pronto Badante to stay connected to their assigned case manager. However, the app contains features available to anyone who downloads it: a map of the older person’s surroundings to show the nearest pharmacies, doctors, grocery shops, and other services; local news; and shortcut commands to emergency numbers (NPO-4). This side project was conceived by Umana Persone, a social enterprise also involved in the scientific committee of Pronto Badante and developed by a small Tuscany tech company.

## 7. Shortcomings

### 7.1. Limited Target Audience and Visibility: ‘The World Remains Outside’

We start by considering the project’s quantitative outcomes over the five-year period from 2016 to 2021 (see Section 4). Of those people who were visited at home, 72% issued a voucher, which points to the case management strategy working towards the goal of enabling a fast solution of home-based care work. Nevertheless, two aspects point to limitations in the project’s reach with its target audience. First, there is no distinct statistical category for the target audience of people aged 65 years or older who have not yet used the INPS voucher, which is why we refer to the proxy of people over 65 with disabilities (122.240 in 2019 [53]) and find that, on average, only 13% of this population called the Pronto Badante hotline. Second, only 32% of those who called the toll-free hotline received a home visit. This points to a lack of visibility of the project among possible beneficiaries. Moreover, if two thirds of those who were aware of the project and called its hotline did not receive a home visit, they might have had the wrong impression about what the project offered, or the project’s criteria for admission were not clear in advance to those who called.

In our interviews with those involved directly with organising and coordinating the project, there was no dissatisfaction voiced about these issues and the outcomes of the project. However, a critique was voiced both by those working for the project and those not working for the project. One case manager was vocal about the limited reach of the project given that its admission criteria were selective and narrow: ‘*the world remains outside, it is not managed*’ (NPO-2). When interviewing members of civil society, we did not mention the project in our request for an interview nor in our topic guides. Instead, we asked them about policy interventions regarding MCWs in Tuscany generally. Nevertheless, all of them brought up Pronto Badante during the interview. They emphasised that the project is not well known despite an adequate information online (TU*) because of the lack of dissemination of information by public institutions and its overall reliance on word-of-mouth to reach its target audience (EA*).

### 7.2. Lacking Pathways to Formalisation and Measures to Cool off the ‘Hot Potato’

Several aspects of the design and implementation of Pronto Badante point to its limited potential to create sustainable change in the sector of migrant care work in Tuscany. Based on its design, we identified three changes the project seeks to implement. First, it is designed to rapidly respond to emerging care needs and provide individualised support synergistically with the public and the third sector. This goal has mostly been reached (see Section 6), albeit not entirely (see Section 7.1). Second, the project has an implicit ideal of formal employment relationships in the sector through the conditionality of the voucher it promotes. Third, its effort to provide training to MCWs suggests a goal of professionalising the sector.

The conditions for formal employment relationships in the family assistance sector are difficult to pin down, but the ways through which families find a (migrant) care worker matter. Most of the recruitment of these workers takes place through word-of-mouth. This means that, if someone the family knows refers a worker to them, the family is likely to employ that person either informally without a contract and registration or formally with the help of an association or trade union office. Since Pronto Badante has no official mechanism of recruiting MCWs, this part of the employment process is up to the family. Although some local organisations who participate in the project have set up databases, word-of-mouth continues to be the main recruitment mechanism. Moreover, once an MCW is recruited, the case manager may organise on-the-job training for the MCW, but there is no official follow-up on whether the family continues to employ this worker after the 30-h voucher expires and whether the employment is formalised. Therefore, there are no data collected on these issues and an assessment of the project’s impact on the formalisation of the sector is impossible. The representative of a local family employers’ association pointed out that the decision to employ someone formally continues to be a personal one and one that is not directly promoted or supported by the region’s projects or policies (EA*).

The professionalisation efforts of the project are also limited. In fact, one of the case managers described the issues of training and accreditation of skills of MCWs as a ‘*hot potato*’ because politicians are apprehensive to tackle it and do not campaign on it (NPO-2). A representative of a migrants’ association noted the missing link between training and employment opportunities for migrant women, and the limited content of the trainings (MA*). In addition, there have been experiments in some municipalities to harmonise the accreditation process, but only a region-wide process would be effective in tackling the problem (NPO-2).

The project’s limited contribution to sustainable change in the sector is connected to issues of its design, available resources, and implementation. One issue concerns the time frames and setting Pronto Badante operates in. It began as a local pilot project and then became an experimental project with an annual evaluation, which meant that the project and its partner organisations were constantly operating in the uncertainty of whether it would continue the following year or not, a perception that was echoed by the civil society interviewees (EA*; TU*; AG*; MA*). When we spoke to the politician and public administrators in charge of overseeing the project, they emphasised the project’s small scale and did not see the objectives of the project as a priority in their policymaking. They stressed that, in comparison to the region’s budget from the Fund for Non-Self-Sufficiency of around 80 million Euro, the project’s budget of two million limited its impact (PA-2c). Indeed, the political will to fundamentally change the ways in which the migrant care work sector is operating seems limited. This could be linked to the perception that the main beneficiaries of such change are female migrant workers. After years of lobbying regional politicians, a representative of a migrants’ association pointed to the limited political representation of female migrants, especially of those without residence permits and enfranchisement, as leading to a lack of interest on the part of politicians to take their concerns seriously (MA*).

Ultimately, the project is designed to provide an emergency response and is thus inherently limited in promoting long-lasting change. However, after seven years of providing a service, it would be worthwhile developing its vision further. The temporary nature of its intervention is a design flaw and forfeits the benefits of the network of connections that has been built over the years. To not consider what happens after the project’s intervention and to people who have used the voucher but are still in need of the services provided by Pronto Badante is a shortcoming that needs to be addressed by regional policymakers. The representative of a local family employers’ association suggested developing a continuation of the project that extends beyond the momentary and temporary support (EA*) and, hence, provides a more sustainable pathway to formalisation and professionalisation of MCWs.

## 8. Conclusions

### 8.1. Core Findings

In response to our research question—*in which ways is the region of Tuscany using social innovation to respond to challenges in LTC provision and to integrate MCWs?*—we found that the region of Tuscany has designed and implemented a project with social innovative qualities to respond to the challenge of care emergencies and the integration of MCWs. It does so by bringing together public and third-sector stakeholders who usually work independently of each other and by making use of an existing voucher programme to promote the formal employment of family assistants (including MCWs). Using expert interviews and a systematic approach to social innovation, we evaluated the Tuscan project Pronto Badante, which is designed to respond to emerging care needs of older persons. We found empirical evidence in line with both the support and critique voiced by the experts we interviewed.

### 8.2. Strengths and Shortcomings

Pronto Badante’s strengths lie in its response to emerging LTC needs not publicly provided in Tuscany and to attempt to integrate MCWs, who are often overlooked as a target group for local interventions and social innovations. To target MCWs and issues related to their employment presupposes knowledge about their contribution to overall LTC provision, which is often impeded by the inexistence of reliable data because of the large share of informal employment in the sector. Even in advanced welfare states, such as Germany, statistics about MCWs are vague at best, and policymakers who already turn a blind eye to the issue have inadequate knowledge upon which to base targeted solutions [54]. Moreover, in countries with significant numbers of MCWs, such as Spain and the UK, innovations do exist but tend to be one-sided, focussing exclusively on particular pathologies (such as dementia) or isolated groups of providers [55]. In contrast, the Tuscan project has addressed both social and technological aspects of innovation. It has realised new forms of relations and collaborations between public social services and social cooperatives and helped overcome the welfare sector’s common silo mentality. It has also experimented with forms of technological assistance that proved their effectiveness during the COVID-19 pandemic. Pronto Badante thus counteracts common pitfalls, such as employing an ICT-based solution while underestimating the human interaction and social capital necessary for patients to accept such innovations, especially in their advanced age [56].

However, the project falls short of reaching its potential beneficiaries and lacks systematic pathways to effectively tackle the informal employment of MCWs. Even though the region of Tuscany is one of the most active Italian regions when it comes to experimenting with innovative interventions in LTC [33], its apparent inclination towards launching innovative projects should not be confused with its actual problem-solving and transformation capacities, as is reflected in the weaknesses of Pronto Badante that we identified. The project failed in its attempt to tackle informal employment in the sector. Evidence from countries worldwide suggests that isolated efforts such as this one are rarely fruitful and that it takes comprehensive and longer-term efforts, usually at the national level and involving many stakeholders, to employ both enabling and punitive approaches towards formalisation [57].

### 8.3. Limitations of the Study and Future Research

Finally, we consider some limitations regarding our study. We only had limited access to documents and data from the project Pronto Badante itself, which resulted in a lack of detail in some respects. In addition, we did not include the perspective of users, neither families benefitting from the project’s intervention, nor family assistants (including MCWs). We sought to include these perspectives with our interviews with migrants’ and employers’ associations staff, but interviews with users could provide a deeper understanding of the perceived success of the project, as well as its ability to bring about change in the lives of older people with care needs and the workers who take care of them. Future research could include users’ viewpoints to provide for a more holistic appraisal of such interventions. Moreover, it could also be very insightful to scholars and policymakers from other regions and countries to view the project from a more longitudinal perspective, providing an in-depth analysis of how to implement and develop projects over time. This could help to avoid common pitfalls and inspire long-lasting change instead of only short-term gain.

### 8.4. Outlook 

These limitations notwithstanding, we believe that our analysis provides valuable insights regarding possible pathways for regional policymaking in LTC. Furthermore, considering the EU’s push for comprehensive LTC solutions within its COVID-19 recovery plans [58], regional governments will have to come up with more innovative projects sooner rather than later. In fact, an Italian civil society coalition of 50 organisations has responded to this push by tabling a draft law in March 2022 to create a national LTC system (for its current status, see: www.pattononautosufficienza.it (accessed on 22 August 2022)). If the law passes, regional governments will be vital in implementing its contents. The promising reform proposal inter alia provides for the integration of existing public services and the formal employment of family assistants, two issues that Pronto Badante already addresses. The project thus showcases possibilities for other regions that are facing very similar challenges, while the impediments to its success should inform the design and implementation of interventions outside of Tuscany, and possibly outside of Italy, going beyond jargon and bringing about substantial change for migrant care workers and care-receivers alike.

## Figures and Tables

**Table 1:** Stakeholders interviewed for the study.

Type of Stakeholder Interviewed (Number of Experts)	Interview Reference
Policymakers and public administration (5)	PA-1(a,b); PA-2(a,b,c)
Non-profit organisation staff (4)	NPO-1; NPO-2; NPO-3; NPO-4
Migrants’ association staff (1)	MA *
Employers’ association staff (1)	EA *
Trade union staff (1)	TU *
Employment agency staff (1)	AG *
**Total: 13**	

* indication for second round interviews.

**Table 2:** Conditions for social innovation—Pronto Badante.

	Condition	*How Pronto Badante Meets Conditions*
1.	Oriented towards exceptional societal challenges/social issues.	*Targets emerging LTC needs not publicly provided and addresses informal labour among family assistants.*
2.	Suggests new solutions.	*Approaches people in need in their own homes and within a short time of the need being identified.*
3.	Creates new configurations of social practices/arrangements.	*Makes formal employment of MCWs a condition of receiving the benefit.*
4.	Overcomes traditional dichotomisation of technological and social innovations.	*Integrates both social (via case manager and voucher) and technological assistance (via app).*
5.	Promotes integration and collaboration/partnership of heterogeneous stakeholders that usually do not co-operate.	*Brings together stakeholders of the public and third sectors who usually do not co-operate.*
6.	Consists of integrated patterns of action.	*Has a clear operating procedure.*
7.	Includes reflective and interdisciplinary approaches.	*Carried out by the project’s scientific committee.*
8.	Is oriented towards the key goal of societal usefulness.	*Delivers services at no cost to beneficiaries and strengthens NPOs.*
9.	Creates sustainable measures.	*Fails to fulfil this condition; for example, design and funding do not allow for sustainable change.*
10.	Creates new growth potentials in terms of regular employment.	*Although benefits are conditional on formal employment of MCWs, the project yields only limited results in terms of growth in formal employment.*
11.	Involves end-users as co-producers of services and products.	*Partially fulfils condition by testing the project’s app with selected participants.*

Source: Authors’ assessment following Heinze and Naegele [25].

## Data Availability

Not applicable.
